# Effect of crude fibre additives ARBOCEL and VITACEL on the physicochemical properties of granulated feed mixtures for broiler chickens

**DOI:** 10.5713/ab.23.0218

**Published:** 2023-11-01

**Authors:** Jakub Urban, Monika Michalczuk, Martyna Batorska, Agata Marzec, Adriana Jaroszek, Damian Bień

**Affiliations:** 1 Department of Animal Breeding, Institute of Animal Sciences, Warsaw University of Life Sciences, Warsaw, 02–786, Poland; 2 Department of Food Engineering and Process Management, Institute of Food Science, Warsaw University of Life Sciences, Warsaw, 02–776, Poland; 3 RETTENMAIER Polska Sp.z o.o, Warsaw, 02-366, Poland

**Keywords:** ARBOCEL, Crude Fibre, Physicochemical Property, VITACEL

## Abstract

**Objective:**

The aim of the study was to evaluate the physicochemical properties (nutrient composition, pH, water content and activity, sorption properties) and mechanical properties (compression force and energy) of granulated feed mixtures with various inclusion levels of crude fibre concentrates ARBOCEL and VITACEL for broiler chickens, i.e. +0.0% (control group - group C), +0.3%, +0.8%, +1.0%, +1.2%.

**Methods:**

The feed mixtures were analyzed for their physicochemical properties (nutrient composition by near-infrared spectroscopy, pH with the use a CP-401 pH meter with an IJ-44C glass electrode, water content was determined with the drying method and activity was determined with the Aqua Lab Series 3, sorption properties was determined with the static method) and mechanical properties (compression force and energy with the use TA-HD plus texture analyzer). The Guggenheim-Anderson-de Boer (GAB) model applied in the study correctly described the sorption properties of the analyzed feed mixtures in terms of water activity.

**Results:**

The fibre concentrate type affected the specific surface area of the adsorbent and equilibrium water content in the GAB monolayer (p≤0.05) (significantly statistical). The type and dose of the fibre concentrate influenced the dimensionless C and k parameters of the GAB model related to the properties of the monolayer and multilayers, respectively (p≤0.05). They also affected the pH value of the analyzed feed mixtures (p≤0.05). In addition, crude fibre type influenced water activity (p≤0.05) as well as compression energy (J) and compression force (N) (p≤0.001) (highly significantly statistical) of the feed mixtures.

**Conclusion:**

The physicochemical analyses of feed mixtures with various inclusion levels (0.3%, 0.8%, 1.0%, 1.2%) of crude fiber concentrates ARBOCEL or VITACEL demonstrated that both crude fiber types may be used in the feed industry as a feedstuff material to produce starter type mixtures for broiler chickens.

## INTRODUCTION

Poultry meat production is mainly based on highly-selected hybrids of chicken broilers reared in the intensive production system [1]. From 1957 to 2005, the growth rate of the chicken broiler increased by more than 400% [2,3]. It is assumed that 85% to 90% of the factors responsible for this is genetic selection, the remainder being attributed to diet [3,4]. The genetic advance in broiler production has contributed to the thinning of intestinal mucosa, consequently increasing the risk of development of enteral diseases caused by gut microbiome impairment (*Salmonella*, *Pasteurellosis*, *Colibacteriosis*, *Newcastle disease*). Avian enteral diseases are the underlying causes of economic losses in intensive poultry production [5,6]. Upsetting the homeostasis of the commensal microbiota, triggered by modifications in feed mixture composition, may lead to disorders in the structure and functions of gastrointestinal tract mucosa, inducing diarrhea and deteriorating broiler productivity [6,7]. Dietary fibre (DF) is an important element of poultry diets. Its application prolongs digesta passage through the upper part of the gastrointestinal tract (GIT) [8] and increases hydrochloric acid (HCl) production [6,9]. In addition, low pH found in this GIT section improves the solubility and absorption of mineral salts [6,10]. Dietary fibre (DF) was defined for the first time in 1953 by Ebeney Hipsley as a term describing non-digestible constituents of the plant cell wall. DF is composed of non-starch polysaccharides (NSP), oligosaccharides and lignin, that are digested and hydrolyzed by enzymes. It can be classified as soluble or insoluble in water [11]. Soluble and insoluble fibres serve various functions during digestion and absorption in the gastrointestinal tract. Broiler chickens fed a compound feed with 3% wheat bran addition (a source of crude fibre) showed an increased relative mass of gizzard and small intestine. Enhanced activities of pancreatic amylase and trypsin have been shown to improve digestibility of feed mixture nutrients [12]. The carbohydrate fraction, including the non-digestible crude fibre, is the key component of a feed mixture, regulating the activity of intestinal microflora in broilers [13,14]. The impact of crude fibre on bacterial populations varies depending on its type. The application dried distilled grains with solubles (DDGS) and wheat grain, as crude fibre sources, in doses of 6% in the starter type diet and 8% in the grower type diet for broiler chickens increased populations of *Selenomonadales*, *Enterobacteriales*, and *Campylobacterales* group bacteria [15]. In turn, 0.5% and 1.0% dietary inclusion of inulin led to an increase in the population of *Bifidobacterium* genus bacteria and a decrease in the population of *Escherichia coli* in colonic digesta [16]. It is believed that to a large extent the positive effects of DF on the body are due to its specific physicochemical properties. The physicochemical properties of crude fibre depend on the type of fibre fraction that builds it [17,18] and affect bulk density, water absorption, fermentability, pH and viscosity of digestive contents, gastrointestinal passage rate of digestive contents, short-chain fatty acid production, and microflora status [18,19]. According to Mateos et al [19], a process that enhances the positive effects of crude fibre on production performance and physiological processes in the bird body is lignification. The resulting lignocellulose is a product composed of carbohydrates (cellulose, hemicellulose) and aromatic polymers (lignin). The physical properties and exact composition of the various lignocellulose fractions depend on the source of the fibre. In this study, two types of crude fibre concentrate additives ARBOCEL and VITACEL were used. The ARBOCEL crude fibre concentrate has a water content of 7.7% and a very high swelling power (800%). It contains: crude fibre (65.3%), non-protein nitrogen compounds (25.1%), total protein (1.0%), crude fat (0.3%), and crude ash (0.5%) (J. RETTENMAIER & SÖHNE GmbH + Co KG). Its recommended dose in feed mixtures for poultry is not higher than 0.8% [21]. ARBOCEL is produced from fresh spruce (*Picea*) trees and differs substantially from traditional dietary fibre sources because it does not bind such nutrients as: crude protein, crude fat, micro- and macroelements, found in a feed mixture. It stimulates the work of intestinal villi, enhances the enzymatic activity of the gastrointestinal tract, is mycotoxin-free, and does not contain soluble fibres [22]. In turn, VITACEL crude fibre concentrate includes functional, nutritive plant fibre and dietary fibres, produced from top-quality raw fruit, vegetable, and cereal materials (J. RETTENMAIER & SÖHNE GmbH + Co KG). This study aimed to analyze the physicochemical parameters of feed mixtures for broiler chickens supplemented with crude fibre concentrates: ARBOCEL and VITACEL.

## MATERIALS AND METHODS

### Materials

The experimental material was a starter type feed mixture (administered since day 1 till day 10 of broiler life) having the following composition: wheat, maize, soybean meal (SBM GMO), oils, monocalcium phosphate, chalk, salt, and calcium carbonate. All groups of feed mixtures were produced using the same technology (76°C, temperature of the granulation process; 2 mm, matrix mesh diameter). Necessary amounts of homogenous feed mixture samples were collected from four different sites of the bag, for each of the performed analyses. In each analysis used a different number of samples from each of the feed groups. The control feed mixture (C) was produced without crude fibre addition, whereas the other feed mixtures (n = 8) were supplemented with crude fibre concentrates: ARBOCEL and VITACEL, according to the scheme presented in Table 1.

### Determination of the proximate chemical composition

Contents of crude protein, crude fat, crude fibre, neutral detergent fiber, and crude ash in the feed mixtures were determined with the near-infrared spectroscopy (NIRS) using a NIR Flex N-500 apparatus (Buchi, Flawil, Switzerland). Determinations were made in 3 replications for each feed mixture sample in a group, after disintegration in an impact mill.

### pH measurement

pH is effectively a measure of the concentration of hydrogen ions in a substance. For pH measurements, 1 g of feed mixture was weighed, added to 9 mL of distilled water, thoroughly mixed [23], and left for 15 min. Then, its pH value was measured using a CP-401 pH meter with an IJ-44C glass electrode (Elmetron, Zabrze, Poland) at 25°C±1°C. The pH-meter was calibrated in buffers of pH 4.00, pH 7.00 and pH 9.00 at 25°C ±1°C. The measurements were made for each feed mixture sample in a group, in 6 replications.

### Determination of water content and water activity

Water content or moisture content is the measurement of the total amount of water contained in the examined product. The water content of the feed mixture samples was determined with the drying method (at 105°C for 3 h), in 2 replications. Water activity is a measure of the availability of water required for biological reactions. It determines the ability of microorganisms to grow. Water activity was determined in 2 replications using the AquaLab Series 3 (Decagon Devices Inc., Pullman, WA, USA) at 25°C±1°C. Measurements included initial water activity (a_w_) and water activity after 3 months, and were conducted under controlled conditions in desiccators at various relative air humidity.

### Determination of water vapor sorption isotherms

The isotherms of water vapor adsorption were determined with the static method based on the measurements of the equilibrium humidity between the analyzed feed mixture samples and the atmosphere with a specific relative humidity [24]. The relative humidity of the atmosphere was controlled using the following saturated salt solutions: LiCl (0.113), CH_3_COOK (0.225), MgCl_2_ (0.328), K_2_CO_3_ (0.423), Mg(NO_3_)_2_ (0.530), NaNO_2_ (0.628), NaCl (0.754), and (NH_4_)_2_SO_4_ (0.810) as well as dry anhydrous CaCl_2_ (0.0). Feed mixture samples were pre-dried in a vacuum dryer at a temperature of 40°C for 48 h. Ca. 1 g of thus prepared material was weighed exact to ±0.00001 g using a Metler AG scale, placed in desiccators containing saturated salt solutions, and stored at a temperature of 25°C±1°C for 3 months. Thymol was placed in desiccators with a relative environment humidity exceeding 0.754 to protect the samples from microflora development. After 3 months, the samples were weighed and their equilibrium water content was computed using formula (1) [25]:


(1) $$u=\left[\frac{d}{c\frac{b}{a}}-1\right]\times 100$$

where: *u*, equilibrium water content (g water/100 g dry matter [DM]); *a*, initial mass of the sample from the desiccator with CaCl_2_ (g); *b*, final mass of the sample, after 3-month storage in the desiccator with CaCl_2_; *c*, initial mass of the sample from the desiccator with a specific solution (g); *d*, final mass of the sample, after 3-month storage in the desiccator with a specified solution (g).

Water vapor adsorption isotherms were described using the Guggenheim-Anderson-de Boer (GAB) equation [26] according to formula (2):


(2) $$u=\frac{u_{m}\times C\times k\times a_{w}}{\left(1-ka_{w})[1+(C-1)ka_{w}\right]}$$

where: *a_w_*, water activity; *u*, equilibrium water activity (g water/100 g DM); *u_m_*, monolayer moisture content (g water/100 g DM); *C* and *k*, GAB model parameters related respectively to the properties of the monolayer and multilayers, dimensionless.

Isotherm approximation was performed based on all measurement points (2 replications). Table Curve 2D software (Jandel Scientific, San Rafael, CA, USA) was deployed to fit the tested GAB model to empirical data.

The goodness of fit of the GAB model to empirical data was evaluated based on the coefficient of determination (R^2^) and root mean square (RMS) error expressed in % [24]:


(3) $$RMS=\sqrt{\frac{\Sigma {\left(\frac{u_{e}-u_{p}}{u_{e}}\right)}^{2}}{N}}\times 100\%$$

where: *u_e_*, experimental equilibrium water content (g water/100 g DM); *u_p_*, experimentally-determined water content (g water/100 g DM); *N*, number of samples.

The specific surface area of feed mixture granules was computed using formula (4) [24]:


(4) $$S=(u_{m}\times N_{o}\times \sigma_{o})/\text{M}$$

where: *S*, specific surface area of the adsorbent (m^2^/g DM); *u_m_*, equilibrium water content in the GAB monolayer (g of water/100 g DM); *N_o_*, Avogadro’s constant (6.023×10^23^ molecules/mol); *σ_o_*, specific surface area of a water molecule (10.6/10^20^ m^2^/molecule); *M*, molecular weight of water (18 g/mol).

### Measurements of mechanical properties

Mechanical properties, i.e., compression force (N) and compression energy (J) necessary to compress feed mixture granules, were measured using a TA-HD plusC texture analyzer (Stable Micro Systems, Godalming, UK). Compression tests of single granules were performed using a probe (20 mm in diameter) at the speed of 2 mm/s. Measurements were carried out in 10 replications.

### Statistical analysis

The statistical analysis of study results was conducted using Statistica 13.1 software. Two-way analysis of variance was deployed to evaluate the impact of crude fibre type (ARBOCEL or VITACEL), crude fibre content, and crude fibre type× content interaction on feed mixture properties. Mean values of the parameters determined in 9 groups of feed mixtures were compared using the Duncan test at a significance level of p≤0.05. The coefficient of Pearson correlation between crude fibre content, compression force and compression energy of feed mixture granules was computed as well.

## RESULTS

### Analysis of the chemical composition of feed mixtures from the analyzed groups

Table 2 presents contents of crude protein, crude fibre, and selected amino acids in the analyzed feed mixtures. The highest total protein content was determined in the feed mixture group C, and the lowest one - in the V1.2 group. The mean crude protein content was at 18.85% in the groups of feed mixtures with the addition of crude fibre concentrate ARBOCEL and 18.97% in those supplemented with VITACEL concentrate. The highest crude fibre content was determined in the feed mixture from group A1.2, and the lowest one - in that from group V0.8. The mean total protein content was at 5.71% in the feed mixture groups with crude fibre concentrate ARBOCEL and 4.33% in those supplemented with VITACEL concentrate. The highest lysine content was found in the feed mixtures from A1.0 group (0.75%), and the lowest one in these from V1.0 group. The mean lysine content reached 0.69% in the feed mixtures from the ARBOCEL group and 0.66% in those from the VITACEL group. The highest and lowest methionine contents were determined for group V0.3 and group V1.2 feed mixtures, respectively. The mean methionine content reached 0.48% in the feed mixture samples from the groups with ARBOCEL and 0.47% in those with VITACEL 0.47%. The highest tryptophan content was assayed in feed mixture groups A1.2 and C, and the lowest one in group V1. Its mean content was at 0.13% in groups of feed mixtures supplemented with ARBOCEL and 0.11% in those supplemented with VITACEL. The scope of analyses included also determinations of percentage contents of the following amino acids: threonine, arginine, valine, isoleucine, and leucine (Table 2). The highest and lowest threonine contents were determined in feed mixtures from group C and group V1.2), respectively. The mean threonine content was found at 0.49% in the groups of feed mixtures with ARBOCEL and 0.42% in those with VITACEL. The highest and lowest arginine contents were determined in feed mixtures from group C and group A0.8, respectively. Its mean content was found at 0.80% in the groups of feed mixtures with ARBOCEL and 0.82% in those with VITACEL. The highest content of valine was determined in feed mixtures from group C and the lowest one in those from group V1. Its mean content was at 0.63% in groups of feed mixtures supplemented with ARBOCEL and 0.55% in those supplemented with VITACEL. The highest content of isoleucine was determined in feed mixtures from group C and the lowest one in those from group A0.8. Its mean content was at 0.61% in groups of feed mixtures supplemented with ARBOCEL and 0.64% in those supplemented with VITACEL. The highest and lowest percentage contents of leucine were determined in feed mixtures from group A1.2 and V1, respectively. Its mean content reached 0.99% in groups of feed mixtures supplemented with ARBOCEL and 0.86% in those supplemented with VITACEL.

### Analysis of pH, water content and water activity of feed mixtures from the analyzed groups

The highest mean pH was measured in the feed mixtures from group V1.2, and the lowest one in those from group A1.0. None of the feed mixtures from any group reached pH = 7, meaning none of them had neutral pH. The study results demonstrated interactions between crude fibre type and content in the feed mixtures. The addition of VITACEL crude fibre concentrate to feed mixtures at levels of 0.8% to 1.2% affected their pH (p≤0.05), compared to the control group and group V0.3 (Table 3).

In turn, the water content of the feed mixtures was found to depend on crude fibre type but not on its inclusion level (Table 3). The feed mixtures with ARBOCEL had a higher water content than those supplemented with VITACEL.

Crude fibre type and content were shown to significantly affect water activity (Table 3). The feed mixtures supplemented with ARBOCEL concentrate exhibited a significantly higher water activity compared to the feed mixtures with VITACEL. The highest and lowest water activity was determined in the feed mixtures A1.0 and V0.8, (Table 3). Interactions (p≤0.001) were found between crude fibre type and content. In the case of ARBOCEL concentrate, increasing its content in the feed mixture to 1.0% caused a significant increase in water activity and its significant decrease in group A1.2. Feed mixture supplementation with 0.8%, 1.0%, and 1.2% of VITACEL caused the water activity to significantly increase compared to group C and group V0.3.

### Analysis of mechanical properties of feed mixtures from the analyzed groups

Table 3 presents results from measurements of force (N) and energy (J) needed to compress feed mixture granules. Granules of the feed mixtures from V1.2 group required the highest compression force and energy. In contrast, the lowest values of these parameters were measured in the case of control feed mixture granules. Crude fibre content of feed mixtures had no effect on their mechanical properties. In turn, crude fibre type affected both the compression force and energy; the VITACEL concentrate made the granules more resistant to compression than ARBOCEL.

### Analysis of sorption properties of feed mixtures from the analyzed groups

After 3-month storage, feed mixtures from all analyzed groups reached a similar water activity (Table 4). In the environment with water activity of a_w_ = 0, the water activity of 0.131 to 0.136 reached by the feed mixtures points to their strong water binding. This water was not removed during drying, and its volume depends on the contents of hydrophilic polymers, protein, and polysaccharides. No differences were observed in the water activity between the feed mixtures supplemented with ARBOCEL and VITACEL fibre concentrates as well as between the feed mixtures with fibre addition and the control feed mixtures. All feed mixtures showed similar water adsorption, and crude fibre content increase did not increase it. The feed mixtures from all groups failed to reach the equilibrium state with the environment. In the environment with water activity of aw = 0.44 and higher, the water activity of the feed mixtures from all analyzed groups was lower than that of the surrounding environment (Table 4).

**Figure 1:**
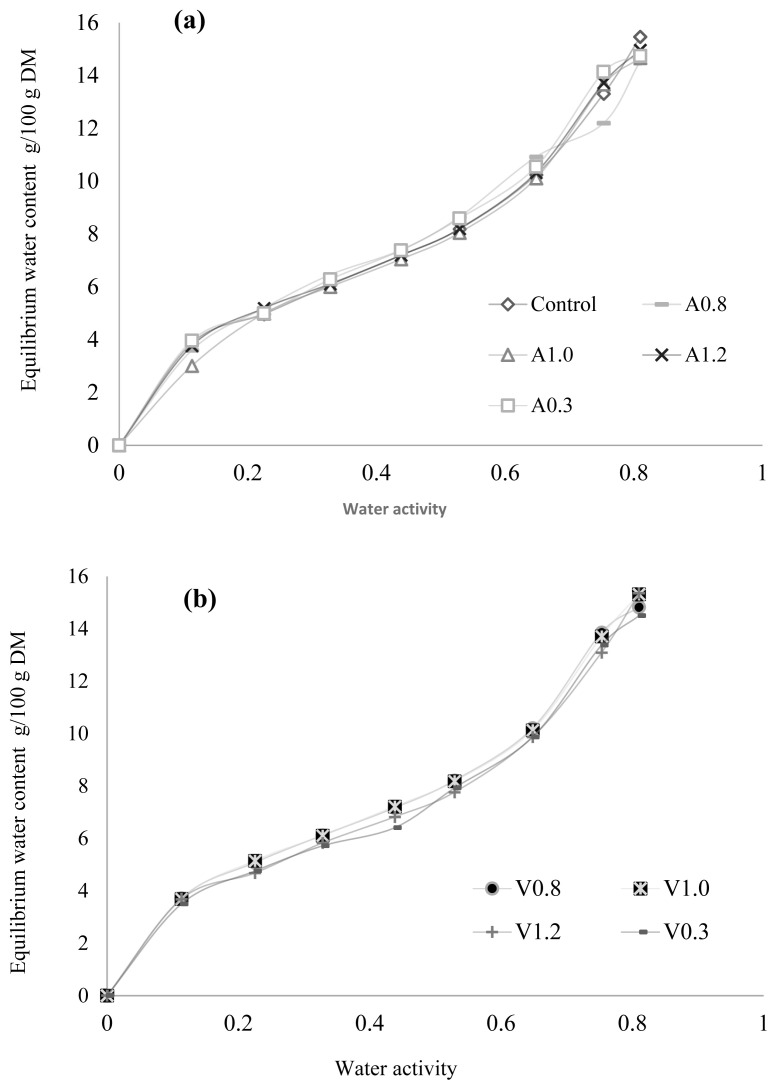
Water vapour adsorption isotherms with different shares of (a) ARBOCEL and (b) VITACEL fibres.

Water vapor adsorption isotherms of the feed mixtures without and with various inclusion levels of ARBOCEL and VITACEL fibre concentrates are presented in Figure 1(a) and 1(b). The shape of all isotherms is typical of materials containing protein and starch. According to the Brunauer Emler Tauler (BET) classification, these are II type isotherms [27]. The course of isotherms was described with the GAB equation, which enabled determining monolayer capacity (water content in the monolayer) (Table 5). The feed mixtures from all analyzed groups had a higher monolayer capacity than the control feed mixture; however, the difference between them was statistically insignificant. Crude fibre type did not affect water content in the monolayer. The feed mixture with VITACEL fibre addition had a higher water content in the monolayer compared to the feed mixture supplemented with ARBOCEL.

The values of the Guggenheim energy constant (C) ranged from 9.9 for the feed mixtures from the control group to 20 for those from V1.2 group, whereas the energy of water binding by feed mixture molecules was very low. The fit of the GAB model to empirical data was expressed by the coefficient of determination (R^2^) and RMS error (Table 5). The highest goodness of fit of the model, determined using R^2^, was demonstrated for the sorption isotherm plotted for A1.2 group and the lowest one - for V0.3 group. The GAB model was found to properly describe sorption data of the analyzed feed mixtures from particular groups in the entire range of water activity values tested.

Parameters of the GAB model were used to estimate the microstructure of the surface of feed mixture granules. The specific surface area of the feed mixture adsorbent was determined based on primary empirical data and determined parameters of the GAB model (Table 5). Its values were affected by crude fibre type, namely: the granules of the feed mixture with VITACEL addition had a significantly greater specific surface area than those of the feed mixture supplemented with ARBOCEL.

## DISCUSSION

### Proximate composition of feed mixture

According to Nutritional Requirements of Poultry (2022), a complete starter type feed mixture should contain 23% of crude protein. In the case of crude protein and total fibre contents, the results obtained in the present study were significantly higher than those reported by the aforementioned author (Table 2). In compliance with nutritional guidelines and recommended nutritional value of feed mixtures for poultry (Nutritional Requirements of Poultry, 2022), the contents of individual amino acids in starter type feed mixtures for broiler chickens should be at: lysine, 1.10%; methionine, 0.50%; tryptophan, 0.20%; threonine, 0.80% arginine, 1.25%; valine, 0.90%; isoleucine, 0.80%; and leucine, 1.20%. The present study results diverged from dietary recommendations for poultry. The percentage contents of the analyzed amino acids were lower than the recommended ones, except for methionine whose content was higher (in Control group) than the recommended level. This discrepancy in results could be due to feed mixture supplementation with crude fibre concentrates. Their increasing percentage content simultaneously decreased the analytical content of the other feed constituents.

### pH, water content and water activity

Unfortunately, there are no published studies among the available literature verifying the effect of the addition of ARBOCEL and VITACEL crude fibre concentrates in feed on pH value, water content and water activity. However, there are publications available that refer to the limits of these parameters and it is these that have been used in the discussion.

The hydrogen ion concentration (pH) in broiler feed is usually close to neutral (pH = 7). It was lower for all feed groups tested, which is more beneficial from the point of view of gastrointestinal tract function, as the lower pH of the feed limits the growth of negative bacterial microorganisms.

Accurate determination of the water (moisture) content of individual feed ingredients and feed mixtures significantly affects the entire feed industry [28]. According to the regulations, the water content of animal feed should not be more than 11.5% [29]. The water content of feed mixtures from all groups analyzed in the present study was lower than the 11.5%.

The occurrence of any changes in the physical, chemical or microbiological properties of feed can lead to a loss of stability. Water activity (a_w_) is one of the most important parameters affecting the stability of feeds used in livestock feeding. Water activity is a measure of the free moisture in a foodstuff. It can also be defined as the quotient of the vapour pressure of a substance divided by the vapour pressure of pure water at the same temperature. The water activity scale ranges from 0 (bone dry) to 1.0 (pure water), but most foods have water activity levels ranging from 0.2 for very dry foods to 0.99 for moist fresh foods [30]. Water activity plays a very important role in microbial stability for both the ingredients used in production and the final animal feed mixture. Water is an essential factor for the growth of bacteria, molds and yeasts; each microorganism has a minimum water activity below which it will not grow [30]. The two most important of these critical values are 0.6 (this is the water activity value at which the growth of any micro-organisms is observed) and 0.86 (this is the lowest value at which the growth of pathogenic bacteria is abolished) [31,32]. The water activity of feed mixtures from all groups analyzed in the present study was lower than the critical values reported by [31,32].

### Mechanical properties

Feed mixture granules are affected by two types of forces: dynamic (associated with feed mixture transport) and static (associated with feed mixture storage). The kinetic and static (hardness) resistance of granules is influenced by the factors resulting from physicochemical traits of raw materials, method of mixture pre-treatment for granulation, and the design of a granulating set, as well as by technical and operational factors. Granule hardness is indicated by the force needed to crush it [33]. The composition of a feed mixture intended for granulation has a key impact on granulation process yield and resistance traits of the granulate. The course of the granulation process is determined mainly by the contents of fat, fibre, protein, and starch in the feed mixture [34]. The high-fibre raw materials positively affect granulate resistance but diminish granulation yield [35]. Dissolved and loosened lignin compounds present in the fibre enhance the impact of cohesive forces during agglomeration. By analogy, the high-fibre granules exhibit high kinetic resistance [34]. Increasing crude fibre content in the feed mixture to 5.74% causes its kinetic resistance to reach 97%, while its further increase causes no statistically significant changes in granulate quality. Increasing fibre content in the granulated material from 2.74% to 18.34% was reported to increase its hardness by 63% on average [34].

### Sorption properties

In general, fibres absorb moisture well and very slowly, with the exception of starch, which does so quickly. The rate of absorption of the fibres depends on their degree of lignification and the intensity of the heat treatment they have undergone. The greater the degree of lignification or the more intense the heat treatment, the slower the absorption rate will be [36].

Lewicki [26] demonstrated that if the value of parameter C exceeded 5.67, the error in mapping the course of multilayer adsorption was lesser than 15.5%. The values obtained in the present study indicate that the GAB model proved very well in describing empirical data, as indicated by the k values (correcting properties of molecules forming the multilayer compared to the liquid phase) ranging from 0.655 to 0.878. The k values of materials whose sorption properties are determined by proteins range from 0.84 to 1.00, whereas these of fibre range from 0.717 to 0.893 [37]. The values of parameters estimated using the GAB model, i.e., water content in the monolayer (u_m_), C (energy constant in Guggenheim equation), and k (constant associated with the energy of interaction between the first and further water molecules adsorbed by individual adsorption centers of the matrix), properly described the sigmoidal shape of the isotherms.

In conclusion, based on the conducted analyses, it was shown that: i) the amount and type of fibre used highly significantly influenced on water activity (however, did not increase the water activity of the tested feed samples above the value of 0.6 (this is the limit above which pathogenic microorganisms begin to develop), which is beneficial for storage conditions) and pH (the addition of crude fiber concentrates did not increase the pH of the tested feed samples above the value of 7 (neutral pH), the use of concentrate additives still maintained the acidic reaction of the feed, which is beneficial for reducing the growth of undesirable microorganisms), the type of fibre used significantly affected the water content (however, it did not increase it above the limit value (11.5%) which brings the benefit of storing finished feed, which does not undergo a faster spoilage process; ii) the type of fibre used highly significantly affected the parameters of the force (N) and compression energy (J) (with the addition of crude fiber concentrate in the feed samples tested, the force and compression energy it took to crush the pellets increased, which in practice means fewer crushed feed pellets lost during the transport of feed from the feed manufacturer to the broiler manufacturer, which means there will be less feed losses); iii) the sorption isotherms of feed samples with the addition of ARBOCEL or VITACEL crude fibre concentrates do not deviate from the sorption isotherms determined for the control feed, which brings the advantage of storing the finished feed, which, not taking up water from the storage environment, does not become spoiled more quickly compared to feeds without the addition of ARBOCEL or VITACEL crude fiber concentrates in their composition.

The results provide answers to many questions that arose before and during the implementation of the experiment, but also bring many additional questions, such as how the addition of ARBOCEL and VITACEL concentrates would affect the other types of feed used in the feeding of broiler chickens (Grower and Finisher) and how the finished feed with the addition of ARBOCEL and VITACEL concentrates would affect the production results, health status and welfare parameters of broiler chickens.

## Figures and Tables

**Table 1:** Experimental scheme - addition of crude fibre concentrates to starter mixtures for broiler chickens

Additive	Groups								

	C	A0.3	A0.8	A1.0	A1.2	V0.3	V0.8	V1.0	V1.2
ARBOCEL (%)	-	0.3	0.8	1.0	1.2	-	-	-	-
VITACEL (%)	-	-	-	-	-	0.3	0.8	1.0	1.2

**Table 2:** Content (%) of crude protein, crude fibre, lysine, methionine, tryptophan, threonine, arginine, valine, isoleucine and leucine in the forage groups studied

Group	CP (%)	CF (%)	Lys (%)	Met (%)	Trp (%)	Thr (%)	Arg (%)	Val (%)	Ile (%)	Leu (%)
C	22.97	5.21	0.73	0.55	0.15	0.56	1.05	0.69	0.70	0.96
A0.3	20.20	4.89	0.68	0.48	0.13	0.49	0.90	0.63	0.65	0.96
A0.8	17.33	5.55	0.59	0.44	0.11	0.41	0.70	0.56	0.53	0.89
A1.0	18.99	5.99	0.75	0.49	0.14	0.52	0.81	0.67	0.62	1.02
A1.2	18.89	6.42	0.73	0.49	0.15	0.53	0.77	0.66	0.62	1.07
V0.3	21.19	4.45	0.67	0.50	0.13	0.49	0.97	0.61	0.66	0.89
V0.8	19.25	3.77	0.69	0.47	0.12	0.42	0.84	0.57	0.65	0.88
V1.0	18.25	3.87	0.56	0.46	0.08	0.39	0.73	0.46	0.60	0.80
V1.2	17.18	5.23	0.73	0.43	0.12	0.38	0.75	0.55	0.66	0.85

CP, crude protein; CF, crude fibre; Lys, lysine; Met, methionine; Trp, tryptophan; Thr, threonine; Arg, arginine; Val, valine; Ile, isoleucine; Leu, leucine.

**Table 3:** Physico-chemical and mechanical parameters of the feed with different additions of ARBOCEL or VITACEL fibre

Item	pH	Water content (%)	Water activity (aw)	Force (N)	Compression energy (J)
Group					
C	6.05^ab^±0.02	8.28^a^±0.06	0.427^b^±0.002	51.37^a^±11.23	6.54^a^±0.38
A0.3	6.04^a^±0.01	9.27^bc^±0.58	0.453^c^±0.010	59.23^ab^±9.74	8.12^abc^±1.55
A0.8	6.13^ab^±0.01	9.35^c^±0.02	0.484^d^±0.004	64.92^bc^±9.35	9.10^bcd^±2.09
A1.0	6.00^a^±0.01	8.66^ab^±0.02	0.487^d^±0.003	63.38^bc^±8.13	8.66^abcd^±2.33
A1.2	6.07^ab^±0.04	8.36^a^±0.33	0.427^b^±0.017	75.28^cd^±8.29	10.76^de^±1.99
V0.3	6.02^a^±0.02	8.19^a^±0.40	0.395^a^±0.005	55.87^ab^±12.94	7.28^ab^±1.28
V0.8	6.11^ab^±0.04	8.19^a^±0.08	0.431^b^±0.004	67.14^bc^±12.14	9.19^bcd^±2.11
V1.0	6.13^ab^±0.01	8.38^a^±0.27	0.437^b^±0.015	66.17^bc^±16.22	9.71^cd^±2.31
V1.2	6.18^b^±0.12	8.22^a^±0.08	0.435^b^±0.003	83.51^d^±13.07	12.86^e^±2.55
p-values					
TF	0.024^*^	0.002^*^	<0.001	<0.001	<0.001
FC	0.005^*^	0.161	<0.001	0.377	0.304
TF×FC	0.020^*^	0.081	<0.001	0.527	0.270

TF, type of fibre; FC, fibre content.

a–e Means with a column significantly different at p<0.05.

* Significance at the p<0.05 level.

**Table 4:** Water activity in the feed mixtures after 3 months storage in an environment providing water activity from 0 to 0.81

Group	Water activity of the surrounding environment								

	0	0.11	0.23	0.33	0.44	0.53	0.65	0.75	0.81

	Water activity of the feed								
C	0.13	0.16	0.30	0.35	0.42	0.50	0.58	0.72	0.76
A0.3	0.13	0.18	0.29	0.35	0.41	0.50	0.60	0.71	0.77
A0.8	0.14	0.19	0.27	0.34	0.42	0.50	0.56	0.70	0.76
A1.0	0.14	0.20	0.28	0.35	0.43	0.49	0.58	0.69	0.77
A1.2	0.13	0.15	0.29	0.35	0.43	0.50	0.59	0.72	0.77
V0.3	0.14	0.21	0.26	0.34	0.41	0.50	0.59	0.70	0.77
V0.8	0.13	0.22	0.28	0.33	0.41	0.49	0.58	0.71	0.77
V1.0	0.13	0.20	0.26	0.35	0.41	0.50	0.59	0.71	0.77
V1.2	0.14	0.14	0.27	0.35	0.41	0.49	0.58	0.71	0.76

**Table 5:** Estimated parameters of water vapour adsorption isotherms according to the Guggenheim-Anderson-de Boer model and sorption area

Item	*u_m_* (g H_2_O/100 g DM)	C	k	R^2^	RMS (%)	S
Group						
C	7.90^a^±0.01	9.90^a^±0.13	0.655^a^±0.052	0.993	9.60	280.1^a^±0.3
A0.3	8.59^ab^±0.18	12.53^ab^±0.62	0.785^b^±0.028	0.992	11.19	304.7^ab^±6.5
A0.8	9.03^abc^±0.07	14.40^bc^±1.09	0.774^b^±0.016	0.996	9.92	320.4^abc^±2.4
A1.0	7.98^a^±0.09	11.95^ab^±0.66	0.807^b^±0.021	0.991	9.75	282.9^a^±3.3
A1.2	9.61^bc^±0.74	17.50^cd^±3.49	0.795^b^±0.014	0.999	9.83	340.9^bc^±26.1
V0.3	8.71^ab^±0.17	14.57^bc^±0.39	0.786^b^±0.001	0.982	11.01	308.9^ab^±5.9
V0.8	9.32^abc^±0.25	16.17^c^±1.56	0.839^b^±0.032	0.991	10.10	330.7^abc^±8.8
V1.0	9.96^bc^±0.88	16.69^c^±0.26	0.878^bc^±0.031	0.998	9.72	353.3^bc^±31.1
V1.2	10.46^c^±1.27	20.02^d^±0.02	0.802^c^±0.021	0.998	8.66	370.9^c^±44.9
p-values						
TF	0.030^*^	0.005^*^	0.040^*^	-	-	0.031^*^
FC	0.062	0.004^*^	0.025^*^	-	-	0.063
TF×FC	0.214	0.497	0.072	-	-	0.214

*u_m_*, monolayer moisture content; TF, type of fibre; FC, fibre content.

a–d Means with a column significantly different at p<0.05.

* Significance at the p<0.05 level.
